# Antibiotic Use in Poultry Production in Grenada

**DOI:** 10.1155/2019/6785195

**Published:** 2019-06-27

**Authors:** Lindonne Glasgow, Martin Forde, Darren Brow, Catherine Mahoney, Stephanie Fletcher, Shelly Rodrigo

**Affiliations:** ^1^Department of Public Health & Preventive Medicine, St. George's University, St. George, Grenada; ^2^Public Health Unit, South Western District Local Health District, Liverpool, Australia

## Abstract

**Objective:**

Research is often lacking in low-income countries to substantiate the regulation of antibiotics in poultry production. Nonregulation of antibiotics in food animal industries has implications for human health. This study was conducted to provide an understanding of farmers' knowledge, attitudes, and practices regarding the use of antibiotics in poultry production in Grenada.

**Method:**

A cross-sectional study was conducted in August-September, 2016, surveying 30 poultry farmers each having 500 or more chickens grown for commercial purposes.

**Results:**

More than 1000 birds were kept on 18 (60.0%) farms. Antibiotics were used on the majority of farms (25, 83.3%). More than half of the respondents, 19 (63.3%), stated they were only somewhat aware of issues related to the use of antibiotics and the majority, 21 (70.0%), were also unable to define antimicrobial resistance. There was inconsistency in the farmers' knowledge about how and when to use antibiotics. There was also a high level of noncompliance with manufacturers' recommendations for use of antibiotics. The respondents were not aware of local programs to monitor antibiotic use or manage antibiotic resistance in the poultry industry.

**Conclusion:**

Generally, the farmers' knowledge and practices were inconsistent with recommendations by the World Health Organization for antibiotic stewardship. While low-income countries, such as Grenada, are challenged with the lack of resources to undertake research and implement responsive actions, this research highlights the need for some immediate measures of remedy, such as education of farmers and monitoring procurement and use of antibiotics, to reduce risk to public health.

## 1. Introduction

The World Health Organization (WHO) identified antibiotic resistance as one major global human health challenge [1]. The indiscriminate use of antibiotics in food animal production is an important factor contributing to the surge of antibiotic resistance [2, 3]. In many low-income countries, antibiotic use in food animals remains unregulated, leading to inappropriate use of the drugs and widespread increase in antibiotic resistance [4]. There is evidence that the lack of education for farmers on the proper use of antibiotics, limited awareness programs, and the widespread dependency on antibiotics are additional factors that contribute to exacerbating this problem [2, 5].

In veterinary practice, antibiotics are administered for treatment of infections, prevention of diseases, and growth promotion. Overuse of antibiotics, however, contributes to the emergence of antibiotic-resistant genes in bacteria. To date, there is very little reliable data on the quantity of antibiotics used in food animal production. There are indications, however, that nontherapeutic use of antibiotics in animals far outweighs its use in humans. In one study, it was estimated that close to 25 million pounds of antimicrobials are used for nontherapeutic purposes in chickens, pigs, and cows whereas only 3 million pounds are used for human medicine [6].

In a study analyzing the bacterial resistance profiles of commercial eggs in Grenada, 65.0% of isolates from the shell membrane and 52.0% from the yolk were resistant to one or more antibiotics [7]. Ampicillin resistance was the most common among isolates from the shell (42.9%) and from the yolk (31.8%), and tetracycline resistance was the second most common. Resistance to three or more antibiotics was found in 10.9% of the isolates, with ampicillin and amoxicillin being the most common among the multidrug resistant isolates. Other studies have also shown that antimicrobial resistance is common in isolates from chickens in Grenada [8–11].

Despite the findings of antimicrobial resistance in poultry, studies were not conducted in Grenada to assess knowledge, attitude, and practices (KAP) related to antibiotic use by poultry farmers. This study was, therefore, conducted to collect baseline data on the use of antibiotics in poultry production and to provide a greater understanding of the potential impact of veterinary practices on public health.

## 2. Materials and Methods

A cross-sectional study was conducted over two months from August to September, 2016, including all poultry farms listed by the Ministry of Agriculture in Grenada with 500 or more chickens grown for commercial purposes. The scope of the study was limited to the mainland in the State of Grenada. Farms in Carriacou and Petite Martinique were not included in the study. A questionnaire was administered to one employee on the poultry farm to collect general information about the farm, use of antibiotics in production, knowledge about antimicrobial resistance (AMR), training and awareness about the proper use of antibiotics, procurement of antibiotics, and monitoring of antibiotics use on the farm. Quantitative data were entered and analyzed in Epi Info 7.2.0.1. Descriptive analysis was conducted to determine the frequency of responses in the survey. Qualitative data were also summarized by themes.

Approval for the study was granted by St. George's University Institutional Review Board. Each respondent was required to provide written informed consent prior to participation in the study.

## 3. Results

### 3.1. Description of Respondents and Poultry Farms

Thirty (30) poultry farms were listed by the Ministry of Agriculture in Grenada having 500 or more chickens grown for commercial purposes. Each of the 30 listed farms participated in the study giving a response rate of 100%.

More than half of the farms, 18 (60.0%), had more than 1000 birds. The majority of farms were located in St. Andrew which is the largest parish on the mainland. None of the farms that had 500 or more birds were located in the parish of St. Mark. Most of the respondents were employed on the farm for 1-10 years.  Table 1 provides a description of the farms and respondents in the study.

### 3.2. Knowledge about Antibiotics and Uses

The majority of the respondents, 27 (90.0%), correctly identified antibiotics as drugs that are prescribed for the treatment of diseases caused by germs. The respondents demonstrated inconsistency in their knowledge about the use of antibiotics in poultry production. Between 4 (13.3%)–22 (73.3%) respondents agreed with incorrect statements about the use of antibiotics in poultry production and 21 (70.0%)–30 (100.0%) agreed with correct statements.  Table 2 shows the responses to statements about the use of antibiotics in poultry production.

### 3.3. Knowledge about AMR

The majority of the respondents, 21 (70.0%), were unable to define antimicrobial resistance. Twenty-five (83.3%) respondents indicated they were not aware of any reports of AMR in the local industry. Only 4 (13.3%) respondents felt that AMR was a problem in the poultry industry in Grenada while half, 15 (50.0%), of respondents did not know whether AMR was a problem, and 11 (36.7%) felt AMR was not a problem.

### 3.4. Training and Awareness about Antibiotic Use

More than half, 19 (63.3%) respondents, reported they were somewhat aware of issues related to antibiotic use in the poultry industry and 10 (33.3%) reported they were very aware.

Respondents also reported that there was a scarcity of education programs on antibiotic use. In the past 3 years, none of the respondents were aware of campaigns on antibiotic use, 26 (86.7%) were not aware of training programs, and 29 (96.7%) were not aware of the distribution of guidelines on antibiotic use. The majority, 18 (60.0%) respondents, stated they never accessed other sources of information such as from the Internet, pharmaceutical representatives, online courses, workshops, medical/veterinary journals, consultations/meetings of the local poultry association, and lectures at academic institutions. The vast majority of respondents, 27 (90.0%), had never received written guidelines from either the Ministry of Agriculture or Ministry of Health. Only 6 (20.0%) respondents reported they received verbal information on antibiotics from an assistant veterinary officer, 3 (10.0%) from chick suppliers in Barbados, and 2 (6.0%) received information from veterinarians at St. George's University.

### 3.5. Poultry Farming Practices related to Antibiotic Use

Twenty-five (83.3%) respondents reported they used antibiotics on their farm while 5 (16.7%) did not use antibiotics. Of the 25 respondents who used antibiotics, 22 (88.0%) reported they used nonprescribed antibiotics. The respondents also reported that antibiotics were used for various purposes and were not restricted to use for specific illnesses. On the 25 farms where antibiotics were used, 4 (16.0%) respondents reported that they used antibiotics as part of the routine feeding cycle, 5 (20.0%) used antibiotics as prophylaxis in young chicks immediately after arrival on the farm, and 12 (48.0%) reported they used antibiotic for any illness or symptoms. Less than half of the respondents, 11 (44.0%), reported they used antibiotics for specific illnesses.

Of the respondents that used antibiotics on their farms, the majority, 20 (80.0%), reported that antibiotics were “prescribed” or recommended for use by themselves or another farm worker, almost half (40.0%) also used antibiotics based on recommendations by a veterinary assistant in the Ministry of Agriculture, and a few used antibiotics based on recommendations from the Internet (1, 4.0%), pharmaceutical agents (1, 4.0%), or chick suppliers (1, 4.0%). Only 3 (12.0%) respondents reported that they used antibiotics based on a prescription from a veterinarian. In most cases, administration of antibiotics on the farm was supervised by the respondents (22, 88.0%) while 6 (24.0%) respondents reported that other farm workers supervised the administration of the drugs. There were no reports of supervision of antibiotics on farms by a veterinarian and only 2 (8.0%) respondents reported supervision by a veterinarian assistant.

Of the 25 respondents that used antibiotics, 24 (96.0%) purchased antibiotics from local agriculture shops. Antibiotics were also purchased from pharmacies by 2 (8.0%) respondents and 4 (16.0%) imported antibiotics from Barbados. The majority, 15 (60.0%) respondents, stored antibiotics in farmhouses and 3 (12.0%) also stored antibiotics in refrigerators in their residence.

Of the 25 respondents that used antibiotics, only 5 (20.0%) frequently consulted with a veterinarian before using the medication, while 13 (52.0%) rarely consulted, and 6 (24.0%) never consulted. Of all respondents, only 2 (6.7%) always used the results from laboratory test to guide their choice of medication to treat their birds while more than half, 18 (60.0%), never used laboratory results and 10 (33.3%) rarely used the results. More than half, 15 (60.0%) of the 25 respondents that used antibiotics reported they had stopped using antibiotics when the symptoms discontinued in the birds and 11 (73.3%) of those that discontinued using further reported that the remaining antibiotics were kept for use in the future. Only 3 (12.0%) of the respondents that used antibiotics reported they had sold meat or eggs within 14 days after using antibiotics.  Table 3 shows the duration of administration of antibiotics by respondents during the lifetime of chickens. Anflox Gold (Norfloxacin), a broad spectrum bactericidal, was most commonly used by the respondents in poultry production. The majority of respondents did not use antibiotics in compliance with manufacturers' recommendations.

### 3.6. Surveillance and Monitoring

A total of 25 (83.3%) respondents were not aware that there was a person or team in the Ministry of Agriculture responsible for infection control. None of the respondents were aware of any programs implemented by the Ministry of Agriculture or Ministry of Health to improve control of antibiotics, monitor antibiotic use, or to manage antimicrobial resistance in the poultry industry.

### 3.7. Recommendations by Respondents to Address Antibiotic Resistance

More than half, 16 (53.3%) respondents, recommended education to address AMR, 10 (33.3%) recommended improving the systems to manage prescriptions and procurement of the drugs, 5 (16.7%) recommended improving farm sanitation and biosafety, and 3 (10.0%) recommended improving the system to monitor antibiotic use by farmers and establishing guidelines for antibiotic use by farmers, respectively.

## 4. Discussion

The increasing emergence of antimicrobial resistant pathogens is a global public health concern. In low-income countries, in particular, antimicrobial use in food animals is not well regulated, contributing to the development and spread of antibiotic resistant organisms [4, 8]. This is the first knowledge, attitude, and practice-based study in Grenada and, as such, the results contribute to providing vital information on the risk factors for antibiotic resistance in a small island state in the Caribbean region. Beyond Grenada, the findings also have implications for public health in other Caribbean islands and internationally through the unlimited movement of people, goods, and animals which provide a ready environment for the spread and multiplication of antibiotic resistant organisms. The findings of this study are, therefore, important to contribute to the development of effective policies and interventions for antibiotic stewardship in poultry production in Grenada and other countries.

The results of this study show farmers were not very knowledgeable about the prudent use of antibiotics in poultry production. While the majority of respondents were able to state the purpose of antibiotics, there was less knowledge about the development and implications of antimicrobial resistance. The majority of respondents also indicated they were only “somewhat” aware of the issues related to antibiotic use in the industry. The low level of knowledge among the respondents is not surprising in this geographical context. Low- and middle-income countries are often more challenged in allocating adequate resources and instituting policies to address the gaps in knowledge and practices in food production industries. Antibiotic use in food animals remains unregulated, leading to inappropriate use of the drugs and widespread increase in antibiotic resistance [4]. Similar findings of low knowledge of antibiotic stewardship have been reported in countries such as in Nigeria [12, 13]. In Nigeria, it was found that the majority of farmers were not aware of nor follow mandatory withdrawal period after administering antibiotics [14], farmers were reportedly unaware of risks from the presence of antibiotic residue in poultry products, and farmers were unaware that misuse of antibiotics is a serious risk to human health. Farmers also agreed that antibiotics were no longer necessary once clinical symptoms subsided [15] and failed to complete the recommended treatment duration [16]. The majority of farmers did not seek veterinary advice for disease diagnosis or an antibiotic prescription but, instead, relied on personal experience, advice from other farmers, or folklore [14]. On the other hand, in most high-income countries, national monitoring programs have been established to control the spread of antibiotic-resistant bacteria [17, 18]. Given the implications of AMR for animal and human health, education and awareness campaigns should be prioritized for poultry farmers in the immediate period to reduce public health risk in Grenada. Further, the ministries of agriculture and health need to institute and enforce regulations, similar to the steps taken in high-income countries, to improve the practices in the poultry industry and, ultimately, safeguard the health of the population.

Given that most respondents were not well informed about best practices in the use of antibiotics, it was not surprising that the producers used antibiotics in a nonprescribed manner that increased the risk of developing AMR in the chickens. Greater risk of exposure to drug-resistant pathogens also increases the potential for transmission to humans through the consumption of poultry products, animal husbandry, and slaughtering [19]. Approximately 130,000 pounds of poultry meat and 24,000 trays of eggs are produced for consumption in Grenada each month [20]. Transfer of AMR pathogens through the food chain and in the environment is an important issue in the context of initiatives undertaken by the Grenada Poultry Association to model the actions undertaken in Barbados to become self-sufficient in poultry meat and egg production. While the move by the Grenada Poultry Association is commendable, the need for antibiotic stewardship becomes even more critical in the sector. Beyond increasing production, the Association must also consider initiatives to improve the food quality in Grenada through the management and control of antibiotics in poultry production.

Despite gaps in knowledge about the prudent use of antibiotic and AMR as well as irregularities in the practices by local poultry farmers, education and awareness programs were virtually nonexistent in the past 3 years. This may be an indication of the Ministry of Agriculture's failure to prioritize addressing the gaps in knowledge and practice or a lack of resources to respond to the situation. Nonetheless, there are legitimate risks to public health, animal health, and the environment in Grenada due to the misuse of antibiotics. AMR is a global issue and, despite their limitations, local and regional organizations must join efforts and strengthen capacities to combat the problem. Academic institutions, such as St. George's University (SGU), can provide invaluable technical support for research and development of the Grenadian poultry industry. Further, regional organizations such as the Food and Agriculture Organization (FAO), Caribbean Agriculture Research and Development Institute (CARDI), Inter-American Institute for Cooperation on Agriculture, and Caribbean Agricultural Health and Food Safety Agency (CAHFSA) can play crucial roles in building capacity and capabilities for managing the use of antibiotics in meat production in Grenada and across the Caribbean region.

More recent literature does not suggest banning all antibiotics in poultry production but limiting the use of nontherapeutic antibiotics which can potentially lead to overuse and the increased prevalence of multidrug resistant pathogens [21]. Most countries in the European Union only allow antibiotic administration under strict veterinary supervision [2]. It is important to identify and assess the factors that contribute to AMR in the Grenadian context, which has been found to be a common problem in both free range and caged chickens [8–11]. This study advances understanding of the factors that contribute to antimicrobial misuse and the results show there is a need for collaboration between the Ministry of Agriculture and the Ministry of Health to assess risk factors in the poultry industry, develop and distribute protocols to monitor the use of antibiotics, and improve AMR surveillance in animals and humans.

Recall bias is a limitation of the study. While respondents were forthright in providing information on the number of days that antibiotic medications were generally administered, most respondents reported that they were unable to recall the proportions used in preparing mixtures for oral administration. Independent assessment of compliance with recommended dosage was not conducted.

## 5. Conclusion

This study is the first comprehensive assessment of the use of antibiotics in the poultry industry in Grenada. Over 200 poultry farms were registered with the Grenada Poultry Association; however, limited resources were available for this study and, therefore, only the larger farms with 500 or more birds were included, assuming that the practices on larger farms could potentially have a greater cumulative impact on food safety in Grenada. This study found several gaps in poultry farmers' knowledge and practices that reflected the need for interventions to achieve objectives of the WHO global action plan on AMR. While small island states are challenged by the lack of resources to undertake research and implement responsive actions, some measure of remedy such as education of poultry producers on the safe and judicious use of antibiotics must be considered immediately to reduce public health risk. Education and awareness for farmers and strengthening monitoring programs can be first steps while measures are put in place to undertake other activities.

## Figures and Tables

**Table 1:** Description of Poultry Farms and Respondents in the Survey.

*Years of farm operation *	n (%)	95% CI

0-10	13 (43.33)	27.37-60.80

11-20	9 (30.00)	16.66-47.88

21-30	5 (16.67)	7.34-33.57

31+	3 (10.0)	3.46 – 25.62

*Parish where farm is located *		

St. Andrew	11 (36.67)	21.88-54.49

St. David	6 (20.00)	9.51-37.31

St. George	4 (13.33)	5.31-29.68

St. John	4 (13.33)	5.31-29.68

St. Patrick	5 (16.67)	7.34-33.57

*Number of years employed with farm *		

*0-10*	15 (50.00)	33.15-66.85

*11-20*	7 (23.33)	11.79-40.92

*21-30*	5 (16.67)	7.34-33.57

*31+ *	3 (10.0)	3.46 – 25.62

*Employee activities on farm *		

Farm hand	30 (100.00)	88.65-100.00

Animal welfare (including administering of medication)	30 (100.00)	88.65-100.00

Slaughtering/meat processing	18 (60.00)	42.32-75.41

*Number of birds on farm *		

500-1000	12 (40.00)	24.59-57.68

1001-2000	6 (20.00)	9.51- 37.31

2001-3000	5 (16.67)	7.34-33.57

3001-4000	4 (13.33)	5.31-29.68

4001-5000	1 (3.33)	0.59-16.67

5001-6000	2 (6.67)	1.85-21.33

*Maximum capacity of farm *		

=<2000	13 (43.33)	27.37-60.8

2001-4000	10 (33.33)	19.23-51.22

4001-6000	3 (10.0)	3.46 – 25.62

6001-8000	1 (3.33)	0.59-16.67

8001-10000	2 (6.67)	1.85-21.33

10001-12000	1 (3.33)	0.59-16.67

**Table 2:** Respondents Responses to Statements About Antibiotics and Uses.

		Agree		*Disagree*		*Neutral*	
		n (%)	95%CI	n (%)	95%CI	n (%)	95%CI
Incorrect Statements	The use of antibiotics in poultry farming is mainly to produce better growth in poultry	16 (53.33)	36.14 – 69.76	8 (26.67)	14.19-44.45	6 (20.00)	9.51- 37.31

	Antibiotics should be added to animal feed at any time to prevent birds from becoming sick.	11 (36.67)	21.88-54.49	16 (53.33)	36.14 – 69.76	3 (10.0)	3.46 – 25.62

	It is necessary to give antibiotics to birds reared in crowded conditions to prevent the spread of disease.	22 (73.33)	55.55-85.81	5 (16.67)	7.34-33.57	3 (10.0)	3.46 – 25.62

	Once a bird shows any kind of symptoms, antibiotics should be given to the birds to prevent the entire flock from becoming infected.	18 (60.00)	42.32-75.41	12 (40.00)	24.59-57.68	-	

	When antibiotics are about to expire, it is better to give medication to the birds to prevent wastage.	4 (13.33)	5.31-29.68	26 (86.67)	70.32-94.69	1 (3.33)	0.59-16.67

Correct Statements	Antibiotics must be placed in a restricted area and accessed only by specific staff when needed.	30 (100.00)	88.65-100.00	-		-	

	Antibiotics should only be prescribed by a veterinary surgeon/veterinarian.	21 (70.00)	51.12– 83.34	9 (30.00)	16.66-77.88	-	

	Antibiotics can be passed to humans from consumption of poultry meat.	26 (86.67)	70.32-94.69	33 (10.0)	3.46 – 25.62	1 (3.33)	0.59-16.67

	Antibiotics can be passed to humans from consumption of eggs from poultry.	24 (80.00)	62.69-90.49	3 (10.0)	3.46 – 25.62	3 (10.0)	3.46 – 25.62

**Table 3:** Duration of Administration of Antibiotics by Poultry Farmers.

Type	Frequency of use in the lifetime of birds	Number of days administered	*∗*Manufacturer's recommended dosage for poultry (http://export.anupco.com/Products)	Compliance (Yes/No)
Anflox Gold (Norfloxacin)	4	1-3 days	12 mg norfloxacin per kg b.w. for 3-5 days.	No

	1-2	5-7 days		Possibly

	1	2 days		No

	2-3	2-3 days		Possibly

	1	7 days		No

	2	2 days		No

	1	5 days		Yes

	1	3-4 days		No

	4	3 days		Yes

	1	5-7 days		No

	2	2-3 days		Possibly

	1	7 days		No

Oxytetracycline (OTC)/OTC Plus			Prevention: 50 g OTC Plus in 45 litres drinking water for 7 days Treatment: 200 g OTC Plus in 45 litres drinking water for 7 days	

	1	5-7 days		Possibly

	1	7 (alternate days)		No

	1	7 days		Yes

	1	7 days		Yes

	2	7-14 days		No

	3-4	6 days		No

	1	2-5 days		No

Chlortetracycline (CTC)			Dissolve 100 g CTC 20% per 150 litres drinking water. Continue treatment for 5-7 days.	

	1-2	5-7 days		Yes

	1	4 days		No

	2-3	2-3 days		No

	2	7 days		Yes

	1	5 days		Yes

	1	20 days		No

## Data Availability

The data used to support the findings of this study are included within the article.

## References

[1] World Health Organization (2015). *Global Action Plan on Antimicrobial Resistance*.

[2] Boamah V., Agyare C., Odoi H., Dalsgaard A. (2016). Antibiotic practices and factors influencing the use of antibiotics in selected poultry farms in Ghana. *Journal of Antimicrobial Agents*.

[3] Ventola C. (2015). The antibiotic resistance crisis: part 1: causes and threats. *Pharmacy and Therapeutics*.

[4] Usui M., Ozawa S., Onozato H. (2014). Antimicrobial susceptibility of indicator bacteria isolated from chickens in Southeast Asian countries (Vietnam, Indonesia and Thailand). *The Journal of Veterinary Medical Science*.

[5] Oluwasile B., Agbaje M., Ojo O., Dipeolu M. (2014). Antibiotic usage pattern in selected poultry farms in Ogun state. *Sokoto Journal of Veterinary Sciences*.

[6] Landers T. F., Cohen B., Wittum T. E., Larson E. L. (2012). A review of antibiotic use in food animals: perspective, policy, and potential. *Public Health Reports*.

[7] Arathy D., Vanpee G., Belot G., Mathew V., Deallie C., Sharma R. (2011). Antimicrobial drug resistance in escherichia coli isolated from commercial chicken eggs in Grenada, West Indies. *West Indian Medical Journal*.

[8] Amadi V., Watson N., Onyegbule O. (2015). Antimicrobial resistance profiles of escherichia coli recovered from feces of healthy free-range chickens in Grenada, West Indies. *International Journal of Current Microbiology and Applied Sciences*.

[9] Eid S., Nasef S., Erfan A. (2015). Multidrug resistant bacterial pathogens in eggs collected from backyard chickens. *Assiut Veterinary Medical Journal*.

[10] Tambur Z., Miljkovic-Selimovic B., Radakovic S., Kulisic Z., Markovic M. (2013). Frequency of antimicrobial resistance in thermophilic campylobacter strains from humans, poultry and pigs. *Vojnosanitetski Pregled*.

[11] Stone D., Davis M., Baker K. (2013). MLST genotypes and antibiotic rResistance of campylobacter spp. isolated from poultry in Grenada. *BioMed Research International*.

[12] Katakweba A., Mtambo M., Olsen J., Muhairwa A. (2012). Awareness of human health risks associated with the use of antibiotics among livestock keepers and factors that contribute to selection of antibiotic resistance bacteria within livestock in Tanzania. *Livestock Research for Rural Development*.

[13] Kim D., Saegerman C., Douny C. (2013). First survey on the use of antibiotics in pig and poultry production in the Red River Delta Region of Vietnam. *Food Public Heal*.

[14] Nsofor C., Olatoye I., Amosun E. (2013). Escherichia coli from Nigeria exhibit a high prevalence of antibiotic resistance where reliance on antibiotics in poultry production is a potential contributing factor. *African Journal of Microbiology Research*.

[15] Adebowale O. O., Adeyemo O. K., Awoyomi O., Dada R., Adebowale O. (2016). Antibiotic use and practices in commercial poultry laying hens in Ogun State Nigeria. *Revue D’Élevage et de Médecine Vétérinaire des Pays Tropicaux*.

[16] Amaechi N. (2014). Evaluation of factors regarding misuse of antimicrobials in poultry and piggery farms in Abia State, Nigeria. *Global Journal of Biology, Agriculture & Health*.

[17] Fletcher-Lartey S. M., Andresen D., Van Hal S. (2016). Comparison of enteric protozoan infections in four Australian hospitals: variable tests and variable results. *Parasitology Open*.

[18] Gaarslev C., Yee M., Chan G., Fletcher-Lartey S., Khan R. (2016). A mixed methods study to understand patient expectations for antibiotics for an upper respiratory tract infection. *Antimicrobial Resistance and Infection Control*.

[19] Rinsky J. L., Nadimpalli M., Wing S. (2013). Livestock-associated methicillin and multidrug resistant staphylococcus aureus is present among industrial, not antibiotic-free livestock operation workers in North Carolina. *Plos One*.

[20] Glasgow L., Forde M., Fletcher S., Keku E. (2017). Compliance with the world organisation for animal health guidelines for poultry production in grenada. *World's Poultry Science Journal*.

[21] Marshall B. M., Levy S. B. (2011). Food animals and antimicrobials: impacts on human health. *Clinical Microbiology Reviews*.

